# Targeting alkaline ceramidase 3 alleviates the severity of nonalcoholic steatohepatitis by reducing oxidative stress

**DOI:** 10.1038/s41419-019-2214-9

**Published:** 2020-01-16

**Authors:** Kai Wang, Chuanjiang Li, Xinxin Lin, Hang Sun, Ruijuan Xu, Qingping Li, Yiran Wei, Yiyi Li, Jianping Qian, Cuiting Liu, Qifan Zhang, Sheng Yu, Zhonglin Cui, Xixin Huang, Bili Zhu, Jie Zhou, Cungui Mao

**Affiliations:** 1 Department of Hepatobiliary Surgery, Nanfang Hospital, Southern Medical University, Guangzhou, Guangdong China; 2 Department of Medicine and Cancer Center, the State University of New York at Stony Brook, Stony Brook, New York, USA; 3 The First Clinical College, Southern Medical University, Guangzhou, Guangdong China; 4 Department of Radiation Oncology, Nanfang Hospital, Southern Medical University, Guangzhou, Guangdong China; 5 Central Laboratory, Southern Medical University, Guangzhou, Guangdong China; 6 Huiqiao Building, Nanfang Hospital, Southern Medical University, Guangzhou, Guangdong China

**Keywords:** Lipidomics, Non-alcoholic steatohepatitis

## Abstract

Overload of palmitic acids is linked to the dysregulation of ceramide metabolism in nonalcoholic steatohepatitis (NASH), and ceramides are important bioactive lipids mediating the lipotoxicity of palmitic acid in NASH. However, much remains unclear about the role of ceramidases that catalyze the hydrolysis of ceramides in NASH. By analyzing the National Center for Biotechnology Information (NCBI) Gene Expression Omnibus (GEO) database, we found that alkaline ceramidase 3 (ACER3) is upregulated in livers of patients with NASH. Consistently, we found that Acer3 mRNA levels and its enzymatic activity were also upregulated in mouse livers with NASH induced by a palmitate-enriched Western diet (PEWD). Moreover, we demonstrated that palmitate treatment also elevated Acer3 mRNA levels and its enzymatic activity in mouse primary hepatocytes. In order to investigate the function of Acer3 in NASH, Acer3 null mice and their wild-type littermates were fed a PEWD to induce NASH. Knocking out Acer3 was found to augment PEWD-induced elevation of C_18:1_-ceramide and alleviate early inflammation and fibrosis but not steatosis in mouse livers with NASH. In addition, Acer3 deficiency attenuated hepatocyte apoptosis in livers with NASH. These protective effects of Acer3 deficiency were found to be associated with suppression of hepatocellular oxidative stress in NASH liver. In vitro studies further revealed that loss of ACER3/Acer3 increased C_18:1_-ceramide and inhibited apoptosis and oxidative stress in mouse primary hepatocytes and immortalized human hepatocytes induced by palmitic-acid treatment. These results suggest that ACER3 plays an important pathological role in NASH by mediating palmitic-acid-induced oxidative stress.

## Introduction

Nonalcoholic fatty liver disease (NAFLD) is becoming a worldwide burgeoning health problem and will probably emerge as the main cause of chronic liver diseases^1^. Nonalcoholic steatohepatitis (NASH) is the critically advanced stage characterized by the coexistence of steatosis with hepatocellular death, inflammation, and fibrosis, its progression leads to cirrhosis and liver cancer^2^. However, understanding of the mechanism of NASH progression is still incomplete. Western diet (WD) rich in saturated, trans, and monounsaturated fatty acids has been suggested to be an important risk factor of NAFLD^3,4^. Palmitic acid is the most abundant saturated fatty acid in serum of nonalcoholic fatty liver (NAFL) patients and it is further increased in NASH patients^5^. Overload of palmitic acid is involved in triggering oxidative stress, leading to hepatocellular death and hepatic inflammation during the progression of NASH^6,7^. Thus, palmitic acid has been suggested to drive the progression of NASH through pro-apoptotic and pro-inflammatory actions.

Emerging studies have implicated ceramides in mediating several pathological effects of palmitic acid in the context of NAFLD. Ceramides are not only the major components of lipid bilayer membrane but also bioactive lipids regulating various biology processes, including cell death, oxidative stress, and inflammation^8^. Oversupply of palmitate has been shown to stimulate ceramide synthesis^9,10^. Lipotoxicity of palmitate that exaggerates NAFLD is partially attributed to the elevation of ceramide species mediating cellular damage^11^. For instance, C_16_-ceramide has been shown to activate mitochondrial pathways of apoptosis^12^. Emerging evidence suggests that increase of C_16_-ceramide causes oxidative stress by impairing mitochondrial function and aggravates hepatocellular injury in NAFLD^13,14^. Interestingly, certain ceramides have been reported to interfere with C_16_-ceramide and probably alter its biological functions. Sandra N. Pinto et al. demonstrated that C_18:1_-ceramide regulates the biophysical properties of lipid bilayer membranes by reducing formation of a gel phase distinctly from C_16_-ceramide^15^; Johnny Stiban et al. reported that C_22_-ceramide may prohibit intrinsic apoptosis by inhibiting C_16_-ceramide-mediated mitochondrial permeabilization^16^. These results may suggest that distinct from C_16_-ceramide, other ceramides may protect hepatocytes against palmitate to alleviate NASH rather than mediate the lipotoxicity of palmitate.

Ceramides are generated via different metabolic pathways including the de novo, catabolic and salvage pathways^8^. The de novo pathway of ceramide synthesis begins with the condensation of palmitoyl-CoA and serine catalyzed by serine palmitoyl transferase (SPT), followed by sequential enzymatic reactions catalyzed by ketodihydrosphingosine reductase, (dihydro)ceramide synthases (CerSs), and dihydroceramide desaturases, respectively. In the catabolic pathways, ceramides are derived from the hydrolysis of sphingomyelins by the action of sphingomyelinases (SMases) or the catabolism of glycosphingolipids. In the salvage pathway, ceramides are synthesized from sphingosine (SPH) and fatty acyl-CoA by CerSs. Upon their generation, ceramides are hydrolyzed by the action of ceramidases encoded by five distinct genes (*ASAH1*, *ASAH2*, *ACER1*, *ACER2*, and *ACER3*)^17^. Recent studies have demonstrated that an increase in the hepatic levels of ceramides due to upregulation of CerSs or SMases is linked to the development and progression of NAFLD^18^, but much remains unclear about the role of ceramidases in regulating ceramides in the context of NAFLD.

In this study, we investigated the role of alkaline ceramidase3 (Acer3) in NASH mice induced by palmitate-enriched Western diet (PEWD). Our investigation discloses pathological roles of ACER3 in mediating oxidative stress in hepatocytes in NASH by preventing palmitic acid from being incorporated into C_18:1_-ceramide.

## Materials and methods

### Mice

All mice were housed under conventional laboratory conditions with a constant room temperature (22 ± 2 °C), humidity level (55 ± 5%), 12-h light:12-h dark cycle, and food (W.F. Fisher & Son; Somerville, NJ, USA) and water available ad libitum. The Acer3 knockout mouse line was generated as described in our previous studies^19^. Briefly, exon 8 of the *Acer3* gene is replaced by the neomycin-resistant gene (*Neo*) cassette. Acer3^+/−^ mice with a mixed genetic background were backcrossed to WT C57BL/6J mice for 16 generations to obtain Acer*3*^+/−^ mice with the sole C57BL/6J genetic background. These heterozygous mice were inbred to generate Acer3^−/−^ mice and their Acer3^+/+^ littermates, which were used for further studies. DNA was isolated from mouse tail clips and subjected to genotyping by PCR described previously^19^. Animal studies were approved by the Institutional Animal Care and Use Committee at Stony Brook University (Stony Brook, NY, USA) and Southern Medical University (Guangzhou, GD, China).

### Murine model of NASH

A Western pattern diet (TD88137, Harlan; Southern Easton, MA, USA) was used to establish a murine model of NASH in 6-week-old C57BL6/J mice as described^20^. The TD88137 diet contained more than 60% saturated fatty acids of total fatty acids. Palmitic acid and oleic acid composed of the highest portions of saturated and unsaturated fatty acids which were 28.9% and 20.9% of total fatty acids, respectively. Six-week-old Acer3^+/+^ and Acer3^−/−^ male mice were fed on the TD88137 diet for 8 weeks to induce NASH before being sacrificed. The livers were removed from mice and divided into pieces that were frozen in Tissue-Tek OCT compound, fixed in buffered formalin, preserved in RNAlater (Qiagen; Valencia, CA, USA), or snap-frozen in liquid nitrogen and stored at −80 °C. Serum alanine aminotransferase (ALT) activity and aspartate aminotransferase (AST) activity were determined using ALT and AST Colorimetric Activity Assay Kit (Cayman Chemical; Ann Arbor, MI, USA) following the manufacturer’s instructions.

### Histologic examination

Liver tissues embedded in paraffin blocks were sectioned and tissue sections were stained with hematoxylin and eosin (H&E) for histologic. Masson’s trichrome (Invitrogen; Grand Island, NY, USA) and Sirius Red (Invitrogen; Grand Island, NY, USA) staining were performed to evaluate hepatic fibrosis. Stained sections were used for scoring under an Imager M2 microscope (Zeiss; Thornwood, NY, USA) in a blind manner. The severity of NASH was evaluated according to steatosis areas, inflammatory foci numbers, and hepatic fibrosis score. Briefly, the area of steatosis was defined as liver parenchyma containing lipid vacuoles and measured by Image J software (NCBI; Bethesda, MD, USA). Inflammatory foci were counted in ×20 field of views, five random fields were scored for each section. Masson’s trichrome and Sirius Red-stained sections were used to score fibrosis as previously described^21^. Briefly, no fibrosis was scored 0, perisinusoidal or periportal fibrosis 1, perisinusoidal and portal/periportal fibrosis 2, bridging fibrosis 3, and cirrhosis, widespread fibrosis, and hepatocyte nodule formation 4. Five random fields were scored for each section.

### Immunohistochemical (IHC) staining and terminal deoxynucleotidyl transferase dUTP nick end labeling (TUNEL) assay

Liver tissue sections were prepared as described above and stained with a cleaved-caspase 3 antibody (Cell Signaling; Beverly, MA, USA; catalog number 9664) or 4-hydroxynonenal (4-HNE) antibody (Abcam; Cambridge, MA, USA; catalog number ab46545) using a Histostain-Plus IHC staining kit (Invitrogen; Grand Island, NY, USA). After counter staining with hematoxylin, cells positive for the cleaved caspase 3 or 4-HNE staining were numerated in five 20× random fields in each tissue section under the Imager M2 microscope in a blind manner. TUNEL assays were performed using TACS® 2 TdT diaminobenzidine kit (Trevigen; Gaithersburg, MD, USA) according to the manufacturer’s instructions, methyl green was used for counter staining. TUNEL-positive cells were numerated as described above.

### Isolation of mouse primary hepatocytes

Six to eight-week-old mice were used for mouse primary hepatocytes isolation by 2-step perfusion according to a published protocol^22^ with slight modification. Briefly, mice were anesthetized by isoflurane inhalant and the abdominal cavity was cut to expose the portal vein. A catheter (27 G feeding needle/round tip) was inserted into the portal vein and a small cut on visceral vena cava was made as an exit for perfusion. Livers were perfused at 8 mL/min with Hank's balanced salt solution (HBSS) for 5 min, followed by Dulbecco's modified Eagle's medium (DMEM) with type I collagenase (100 CDU/ml) (Worthington-biochem; Lakewood, NJ, USA) for another 5 min. Digested liver tissues were collected and liver sac was cut to release the hepatocytes, which were passed through cell strainers into 50-ml tubes. Hepatocytes were collected by centrifugation at 50 × *g* for 2 min, washed twice with DMEM medium supplemented with penicillin, streptomycin, and 10% fetal bovine serum (FBS) (Sigma-Aldrich; St. Louis, MO, USA), and resuspended in the same medium. Cell number was counted and cell viability was assessed by Trypan Blue extrusion. Cell viability was maintained at 80–85% for each independent experiment. Hepatocytes (2 × 10^4^ cells/cm^2^) were seeded in cultural plates coated with type I collagen (BD biosciences; Franklin Lakes, NJ, USA) and cultured in the DMEM medium. At 24 h after seeding, hepatocytes were treated with palmitate (Sigma-Aldrich; St. Louis, MO, USA).

### Free fatty acid (FFA)/bovine serum albumin (BSA) complex preparation

FFA/BSA complex was prepared as described^23^ with slight modification. Briefly, 100 mM of palmitate (Sigma-Aldrich; St. Louis, MO, USA) or other FFA was prepared in 0.1 m NaOH at 70 °C. In an adjacent water bath at 55 °C, a 10% (wt/vol) FFA-free BSA (Fisher BioReagents; Pittsburg, PA, USA) solution was prepared in DMEM medium. The FFA solution was added dropwise to the BSA solution at 55 °C, and the FFA/BSA mixture was vigorously vortexed for 10 s before a further 10-min incubation at 55 °C. The FFA/BSA complex solution was cooled to room temperature and sterilized by filtration with a 0.45-μm pore size membrane filter. Prepared FFA/BSA complex was stored at −20 °C.

### ACER3 knockdown in immortalized human hepatocyte L02 cells

Immortalized human hepatocyte L02 cell line^24,25^ purchased from Cell Bank of Shanghai Institute of Biochemistry and Cell Biology in Chinese Academy of Science (Shanghai, China) was grown in DMEM medium containing penicillin, streptomycin, and 10% FBS. A control shRNA (shCON), the first (shACER3-1, CCGGTATACAGCTGTTGCATATTTGCTCGAGCAAATATGCAACAGCTGTATATTTTTTG) and the second (shACER3-2, CCGGCCTCCAATGTTCGGTGCAATTCTCGAGAATTGCACCGAACATTGGAGGTTTTT) ACER3-specific shRNA were purchased from Sigma-Aldrich at St. Louis, MO, USA. One day before transfection, 2 × 10^5^ cells were seeded onto six-well plates. Cells were transduced with lentiviruses expressing shCON, shACER3-1, or shACER3-2. At 48 h post transfection, cells were replated at a 1:100 dilutions and cultured in DMEM with 5 μg/ml puromycin (Sigma-Aldrich; St. Louis, MO, USA) for 2 weeks. Puromycin-resistant clones were selected and expanded. ACER3 knockdown efficiency was examined by real-time PCR (qPCR) analyses and alkaline ceramidase activity assay as following described. 2 × 10^4^ cell/cm^2^ were replated and treated with palmitate 24 h later.

### Cell viability determination

Cell viability was determined using an in vitro toxicology assay kit based on 3-(4, 5-dimethylthiazol-2-yl)-2, 5-diphenyltetrazolium bromide (MTT) (Sigma-Aldrich; St. Louis, MO, USA) according to the manufacturer’s instructions.

### Dihydroethidium (DHE) staining

DHE staining was performed as described^26^. Primary hepatocytes and L02 cells were washed twice with PBS, incubated with 0.5 μM DHE at 37 °C for 30 min, and subsequently washed twice with ice-cold PBS before being observed under a confocal microscope with the excitation wavelength set at 505 nm and emission wavelength at 610 nm (Leica; Chicago, IL, USA). The fluorescence intensity of stained cells was measured with a fluorescence plate reader with the excitation and emission wavelengths set at 505 and 610 nm, respectively.

### Oil red O (ORO) staining

ORO staining of liver tissue sections was performed as described^27^. Briefly, fresh tissues were embedded in Tissue-Tek OCT compound and sectioned into 12-μm-thick sections. After air dry at room temperature for 10 min, sections were incubated with ORO solution (0.375%, wt/vol) (Sigma-Aldrich; St. Louis, MO, USA) for 5 min and then washed for another 30 min in running tap water. Stained sections were mounted on a water-soluble mounting medium and imaged under the Imager M2 microscope in a blind manner. Captured Images were analyzed using the NCBI Image J software (NCBI; Bethesda, MD, USA). ORO staining of cells was performed as described^28^. At 24 h post palmitate treatment, primary hepatocytes and L02 cells were washed twice with PBS and then fixed with 10% formalin for 15 min. Fixed cells were treated with 0.5% ORO for 1 h before being washed several times with distilled water. The stained cytoplasmic neutral lipids were visualized and imaged under an EVOS cell imaging system (Invitrogen; Grand Island, NY, USA). Finally, ORO was solubilized from stained cells with isopropanol and its optical density was measured by spectrophotometry at an absorbance wavelength of 510 nm.

### Protein extraction and immunoblotting

Liver tissues, primary hepatocytes, and L02 cells were lysed in lysis buffer (50 mM Tris–HCl pH 7.5, 250 mM NaCl, 1 mM EDTA, 1 mM EGTA, 1% Triton X-100, 1% SDS) supplemented with protease inhibitor mixture (Roche; Indianapolis, IN, USA) and phosphatase inhibitor cocktail (Thermo Scientific; Waltham, MA, USA). Proteins were separated on SDS–polyacrylamide gels and transferred onto nitrocellulose membranes, which were then incubated with an antibody against caspase 3 (Cell Signaling; Beverly, MA, USA; catalog number 9662), cleaved-caspase 3 (Cell Signaling; Beverly, MA, USA; catalog number 9664), poly(ADP-ribose) polymerase (PARP) (Cell Signaling; Beverly, MA, USA; catalog number 6704), 4-HNE (Abcam; Cambridge, MA, USA; catalog number ab46545), and β-Actin (Santa Cruz; Dallas, TX, USA; catalog number sc-8432).

### Protein concentration determination

Protein concentrations were determined with BSA as a standard using a bicinchoninic acid (BCA) protein determination kit (Thermo Scientific; Waltham, MA, USA) according to the manufacturer’s instructions.

### Alkaline ceramidase activity assays

Alkaline ceramidase activity assays using nitrobenzofurazan-C_12_-phytoceramide as a specific substrate of ACER3/Acer3 were performed as our previous study^29,30^. Briefly, cell membranes of liver tissues or hepatocytes were harvested by ultra-centrifugation and subjected to enzymatic assays. The reaction mixtures were spotted onto a thin-layer chromatography (TLC) plate and developed in a solvent system. The TLC plate was dried and scanned by an imaging system (Typhoon FLA 7000, GE Healthcare Life Sciences; Pittsburgh, PA, USA) setting at fluorescence mode. The fluorescent band of NBD-C_12_-FFA released from nitrobenzofurazan-C_12_-phytoceramide was identified according to the standard NBD-C_12_-FFA spotted on the same TLC plate. The content of NBD-C_12_-FFA in each reaction was determined according to a standard curve generated from known concentrations of the standard NBD-C_12_-FFA.

### Liquid chromatography tandem mass spectrometric (LC–MS/MS) analysis of sphingolipids

Liver tissues, primary hepatocytes, and L02 cells were collected and washed with buffer (25 mM Tris–HCl, pH 7.4, 150 mM NaCl). Lipids from tissue homogenates (2 mg protein per sample) and cells were extracted with ethyl acetate/isopropanol/water (60/30/10, v/v/v). The lipid extracts were dried under N_2_ gas stream and reconstituted in methanol. Sphingolipids were determined by LC–MS/MS, which was performed on a TSQ 7000 triple quadruople mass spectrometer (Thermo Finnigan; Ringoes, NJ, USA) in the Lipidomics Core Facility at Stony Brook University or Southern Medical University as described^31^. Amounts of ceramides were normalized to protein concentration for each individual samples.

### RNA extraction and real-time polymerase chain reaction (qPCR) assays

Total RNA was extracted from liver tissues, L02 cells, and mouse primary hepatocytes using a RNeasy mini kit (Qiagen; Valencia, CA, USA). RNA was reversely transcribed into cDNA and subjected to qPCR analysis. The expression of mRNA for the mouse *Acer3* gene, human *ACER3* gene, mouse interleukin 6 (*Il-6*), mouse tumor necrosis factor-α (*Tnf-α*), mouse transforming growth factor-β (*Tgf-β*), mouse or human β-Actin were measured by qPCR with corresponding primers (Supplementary Table S1). qPCR was done on an ABI Prism 7000 sequence detection system (Thermo; Ringoes, NJ, USA), and results were analyzed with the ABI Prism 7000 software (Thermo; Ringoes, NJ, USA). The mRNA levels of a gene of interest were calculated by the delta–delta CT method using the mouse or human *β-Actin* gene as the reference gene.

### Data analysis

Statistical analyses by a Student’s *t*-test or one-way ANOVA were performed using Statistical Product and Service Solutions software 20.0 (IBM; Armonk, NY, USA). *p*-values < 0.05 were considered significant.

## Results

### Hepatic ACER3/Acer3 is upregulated in patients or mice with NASH

**Fig. 1:**
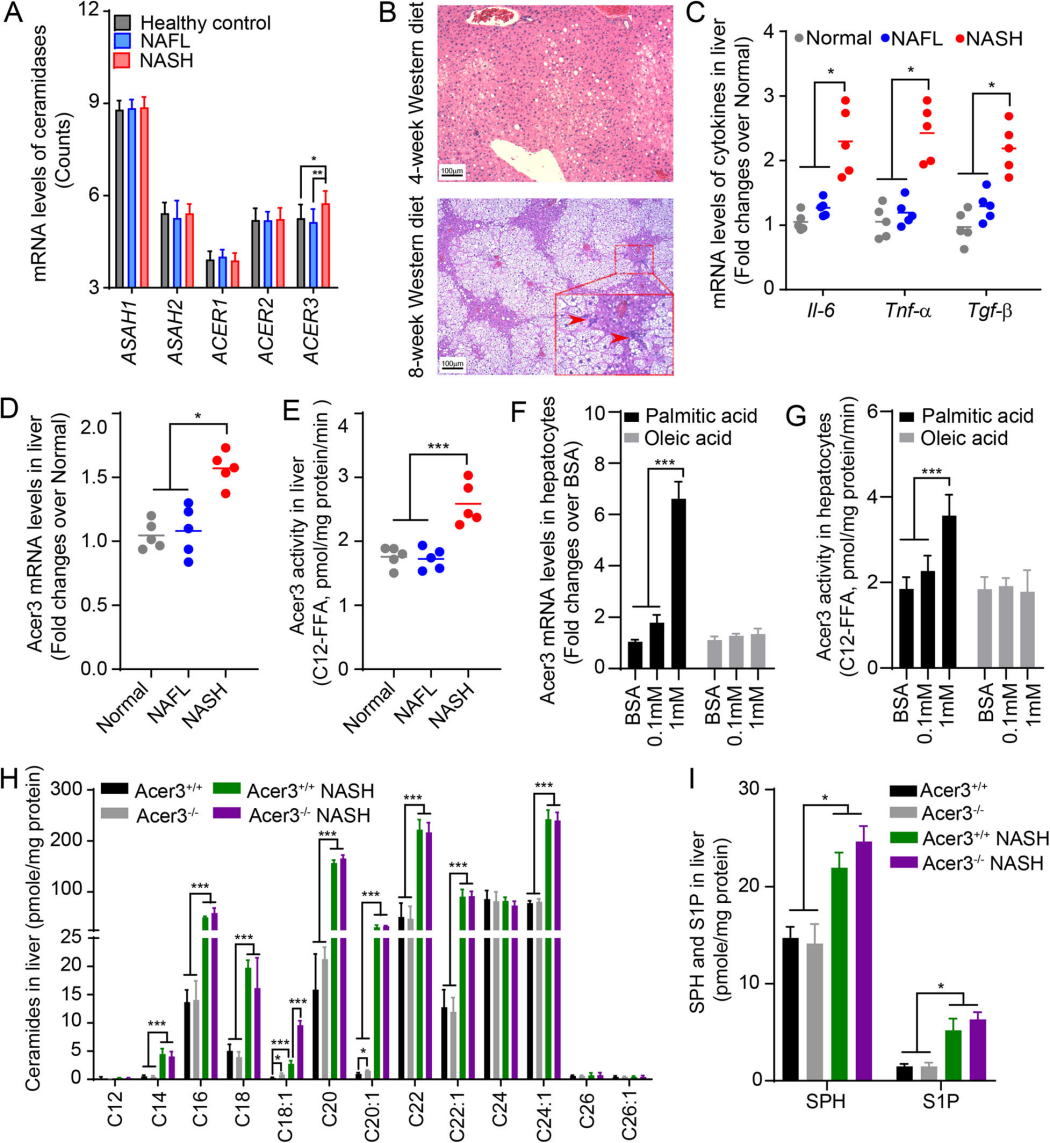
ACER3 regulates the catabolism of hepatic C_18:1_-ceramide in the context of NASH. **a** The NCBI GEO database, GSE48452, was analyzed for mRNA levels of genes encoding sphingolipid-metabolizing enzymes in liver tissues from 14 healthy individuals, 14 NAFL patients, and 18 NASH patients. The mRNA levels of ceramidases, including *ASAH1*, *ASAH2*, *ACER1*, *ACER2*, and *ACER3*, were reported. **b** and **c** 6-week-old C57BL/6J mice were fed standard chow (Normal) or palmitic-acid-enriched WD for 4 (NAFL) or 8 weeks (NASH) before mouse liver tissues were dissected. Livers were subjected to histological analyses for steatosis and inflammatory infiltration **b** or to qPCR analyses for the mRNA levels of *Il-6*, *Tnf-α*, *Tgf-β*, and *β-actin* as a reference gene **c**. Inflammatory foci were marked by arrowheads. **d** and **e** Mice were fed standard chow (Normal) or PEWD for 4 (NAFL) or 8 (NASH) weeks before livers were subjected to qPCR analysis for Acer3 mRNA levels **d** or ACER3 enzymatic activity assays **e**. **f** and **g** Primary hepatocytes were isolated from wild-type mice fed standard chow then treated with fatty acid-free bovine serum albumin (BSA), BSA–palmitate or BSA–oleic acid complex at indicated concentrations. At 6 h post treatment, total RNA and membranes were isolated from hepatocytes and assayed for Acer3 mRNA levels **f** and enzymatic activity **g**, respectively. **h** and **i** Liver tissues were collected from Acer3^+/+^ and Acer3^−/−^ mice fed on standard chow or PEWD for 8 weeks and the hepatic levels of ceramides **h**, SPH, and S1P **i** were determined by LC–MS/MS. Images in **b** represent results from one of five pairs of mice. Data in **d**–**g** represent mean ± SD of three independent experiments. Data in **h** and **i** represent mean ± SD, *n* = 3. *P < 0.05, **P < 0.01, ***P < 0.001.

Increased ceramides have been implicated in the development and progression of NASH^18^, but much remains unknown about the role of ceramidases in NASH. To this end, we first determined if any ceramidase was dysregulated in liver during NAFLD progression by data-mining the NCBI GEO database. In silico analyses using a dataset of gene expression in liver from NAFLD study by Markus Ahrens et al. ^32^ revealed that among the five ceramidase genes, only *ACER3* mRNA levels were significantly upregulated in NASH liver compared to healthy liver and NAFL liver (Fig. 1a). In addition to *ACER3*, the mRNA levels of enzymes involved in the generation of ceramides, including *DEGS1*, *SMPD2*, *SMPD3*, and *SMS2*, were also significantly altered in livers of NAFL or NASH (Supplementary Fig. S1).

To investigate whether *ACER3* upregulation plays a role in the pathogenesis of NASH, we tested whether the mouse *Acer3* gene is also upregulated in liver in a mouse model of NASH. To this end, we induced NASH in Acer3 knockout mice and their WT littermates by feeding mice on PEWD as described^20^. We found that mice developed NAFL after 4-week PEWD feeding, which was characterized with hepatic steatosis (Fig. 1b) without significant inflammation (Fig. 1b, c). Thereafter, NAFL progressed to NASH in mice after 8-week PEWD feeding, characterized with advanced steatosis, inflammatory cell infiltration (Fig. 1b), and elevation of pro-inflammatory cytokines, including *Il-6*, *Tnf-α*, and *Tgf-β* in liver (Fig. 1c). We found that Acer3 mRNA level and its enzymatic activity were not changed in liver tissues from NAFL mice, but were significantly upregulated in liver tissues from NASH mice (Fig. 1d, e). To further elucidate whether Acer3 was upregulated in hepatocytes upon high fat supplement, we tested if expression levels of Acer3 in mouse primary hepatocytes were altered by treatment of palmitate and oleic acid that were the major fatty acids of PEWD. The results revealed that oversupply of palmitate significantly upregulated Acer3 mRNA levels and enzymatic activity in mouse primary hepatocytes, but oleic acid at the same concentration did not (Fig. 1f, g). In conclusion, upregulation of Acer3 in liver with Western-diet-induced NASH is probably dependent on overload of palmitate.

### Acer3 upregulation prevents buildup of C_18:1_-ceramide in NASH liver

To determine if upregulation of Acer3 affected ceramide metabolism in NASH liver, we measured the levels of ceramides in liver tissues collected from the above mice by LC–MS/MS analysis. We found that various ceramides were elevated in NASH liver tissues (Fig. 1h) and that Acer3 deficiency specifically augmented the increase of C_18:1_-ceramide but not other ceramide species, SPH, or sphingosine-1-phosphate (S1P) (Fig. 1h, i). These results suggest that upregulation of Acer3 prevents C_18:1_-ceramide from being accumulated in the mouse liver with NASH.

### Acer3 deficiency attenuates early inflammation and fibrosis without affecting steatosis in the mouse model of NASH

**Fig. 2:**
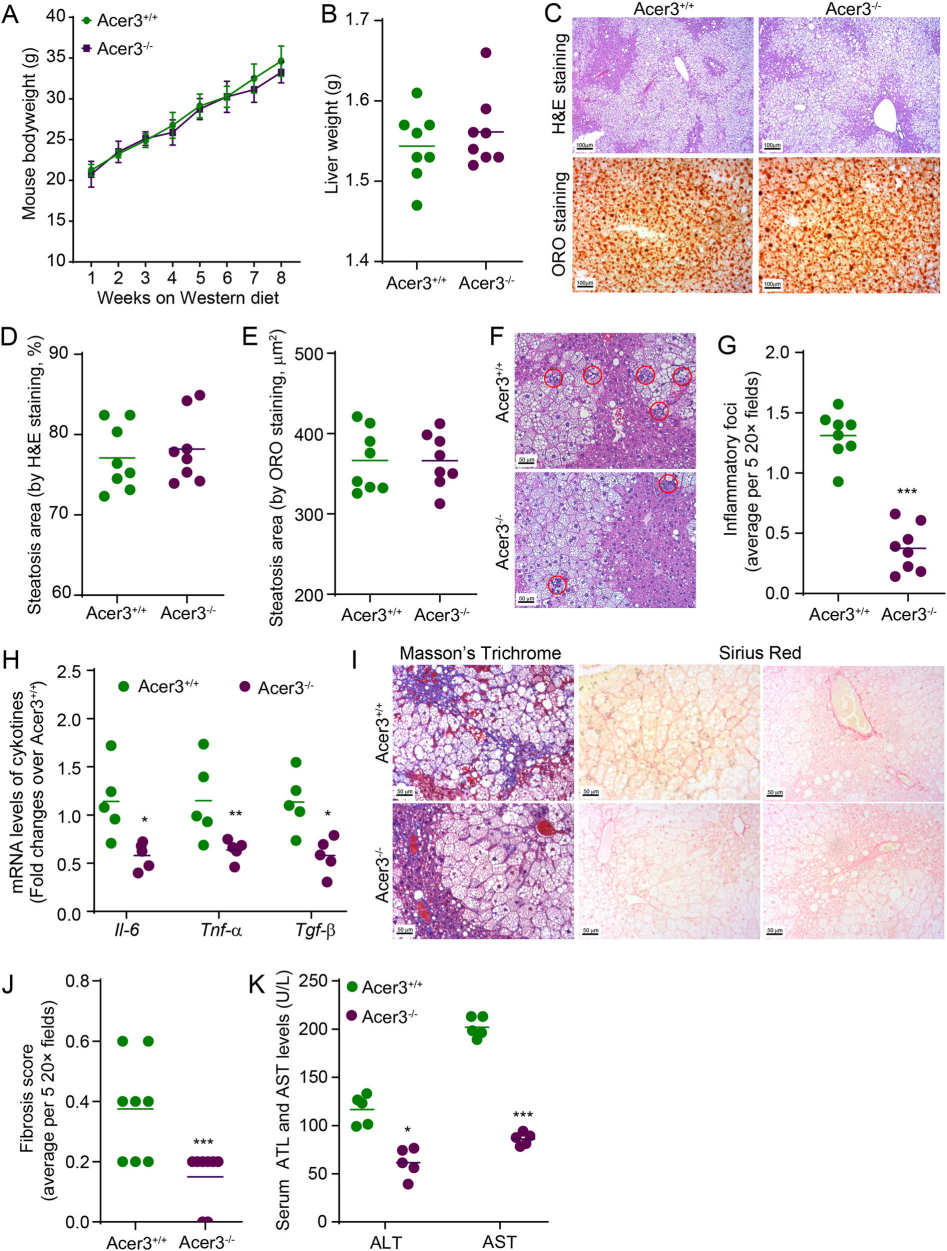
Acer3 deficiency attenuates early inflammation and fibrosis in the liver of mice with NASH. **a** and **b** Acer3^+/+^ and Acer3^−/−^ mice were fed PEWD for 8 weeks and their body weights were recorded weekly **a**. At week 8, mice were euthanized and their liver weights were measured **b**. The liver tissues were subjected to the following assays. **c**–**e** Liver tissues were sectioned for H&E and ORO staining **c**. Liver sections stained with H&E **d** or ORO **e** were analyzed for steatosis areas as described in “Materials and methods” section. **f** and **g** Liver sections were stained with H&E **f** and hepatic inflammatory foci that marked by red cycle were counted to evaluate inflammatory infiltration **g**. **h** Total RNA was extracted from liver tissues and subjected to qPCR analyses for the mRNA levels of *Il-6*, *Tnf-α*, *Tgf-β*, and *β-Actin* as a reference gene. **i** and **j** Liver sections were stained with Mason’s trichrome and Sirius Red **i** and fibrosis was scored as described in “Materials and methods” section **j**. **k** Whole blood was collected from Acer3^+/+^ and Acer3^−/−^ mice fed PEWD for 8 weeks and serum levels of ALT and AST were determined to evaluate hepatocellular injury. Data in **a** represent mean ± SD, *n* = 8. Images in **c**, **f**, and **i** represent results from one of eight pairs of mice. *P < 0.05, **P < 0.01, ***P < 0.001.

Ceramides have been implicated in the development and progression of NAFLD^18^. However, our previous study demonstrated that Acer3 deficiency did not cause overt hepatic abnormalities in mice fed a standard chow^19^. We wondered whether Acer3 upregulation has any impact on NASH development and progression. To this end, we challenged the Acer3 null mice and their wild-type littermates with PEWD, and examined if Acer3 deficiency affected pathogenesis of NASH in terms of steatosis, inflammatory infiltration, and fibrosis. During the PEWD feeding, Acer3^−/−^ and Acer3^+/+^ mice had a similar weight gain (Fig. 2a) and their liver weight was not differed (Fig. 2b). H&E and ORO staining failed to find any significant difference in the area of steatosis in liver between Acer3^−/−^ and Acer3^+/+^ mice (Fig. 2c, e), indicating that Acer3 deficiency does not impact the diet-induced hepatic steatosis. However, H&E staining revealed that the number of inflammatory foci was significantly reduced in the livers from Acer3^−/−^ mice compared to the livers from Acer3^+/+^ mice (Fig. 2f, g). qPCR analyses demonstrated that the mRNA levels of *Il-6*, *Tnf-α*, and *Tnf-β* were significantly lowered in Acer3^−/−^ livers than in Acer3^+/+^ livers (Fig. 2h). These data suggest that Acer3 deficiency reduces inflammation in NASH liver. Masson’s trichrome and Sirius Red staining showed that Acer3^+/+^ livers developed perisinusoidal and even bridging fibrosis, whereas Acer3^−/−^ livers exhibited minor fibrosis that was restricted to the portal area (Fig. 2i). Fibrosis scores in Acer3^−/−^ livers were significantly lower than those in Acer3^+/+^ livers (Fig. 2j), suggesting that Acer3 deficiency inhibits PEWD-induced liver fibrosis. Enzymatic activity assays revealed that Acer3 deficiency significantly decreased the serum levels of ALT and AST in NASH mice (Fig. 2k), indicating that loss of Acer3 indeed attenuates hepatocellular injury in NASH mice. Taken together, these data suggest that Acer3 deficiency alleviates early inflammation and fibrosis without affecting steatosis in NASH mice.

### Acer3 deficiency protects hepatocytes against apoptosis in NASH liver

**Fig. 3:**
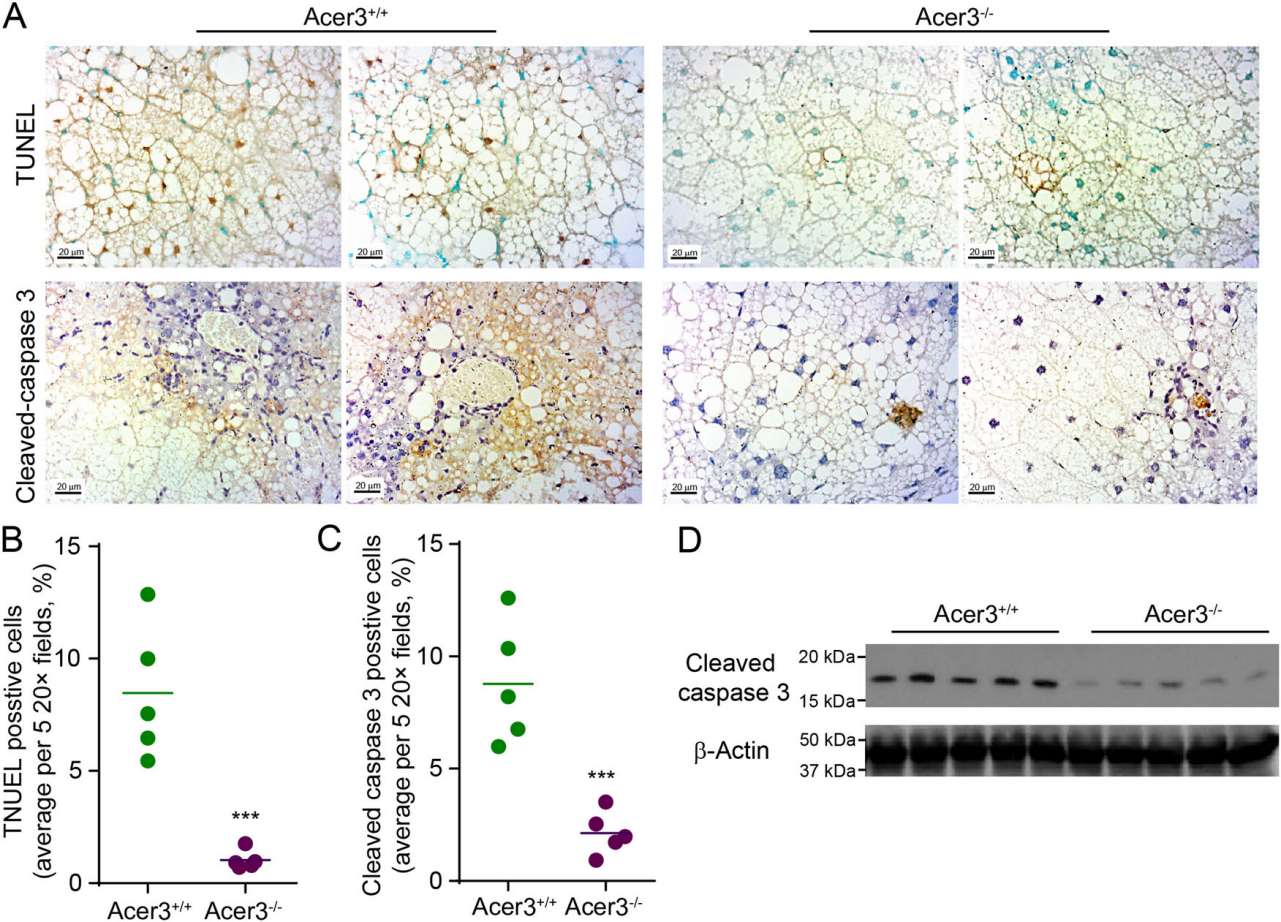
Acer3 deficiency alleviates apoptosis in hepatocytes in mice with NASH. **a**-**c** Liver sections from Acer3^+/+^ and Acer3^−/−^ mice fed PEWD for 8 weeks were stained with TUNEL or a cleaved caspase 3 antibody **a**. Cells stained positive for TUNEL **b** and cleaved caspase 3 **c** were counted to evaluate hepatocellular apoptosis induced by NASH. **d** Liver tissues from the above mice were subjected to Western blot analyses with antibodies against cleaved caspase 3 or β-Actin (a protein loading control). Images in **a** represent results from five pairs of mice. ***P < 0.001.

Death of hepatocytes plays a centric role in mediating inflammation and fibrosis during the progression of NASH^33^. Our previous studies have demonstrated that ACER3/Acer3 regulates cell death and survival by catalyzing the hydrolysis of unsaturated-long-chain ceramides^19,29,30^. Having demonstrated that Acer3 deficiency attenuated inflammation and fibrosis in NASH liver, we examined if Acer3 knockout affected hepatocellular death during NASH development. TUNEL assays revealed a decrease of TUNEL-positive hepatocytes in NASH livers from Acer3^−/−^ mice compared to Acer3^+/+^ mice (Fig. 3a, b), suggesting that Acer3 deficiency inhibits hepatocellular death in NASH liver. Immunostaining found that loss of Acer3 significantly reduced the number of hepatocytes stained positively for cleaved caspase-3, an apoptosis marker, in NASH livers (Fig. 3a, c). Consistently, western blot analysis demonstrated that Acer3 knockout significantly inhibited an increase in the protein levels of cleaved caspase 3 in NASH livers (Fig. 3d). These data suggest that Acer3 deficiency protects hepatocytes from apoptosis in NASH liver.

### Acer3 deficiency reduces oxidative stress in NASH liver

**Fig. 4:**
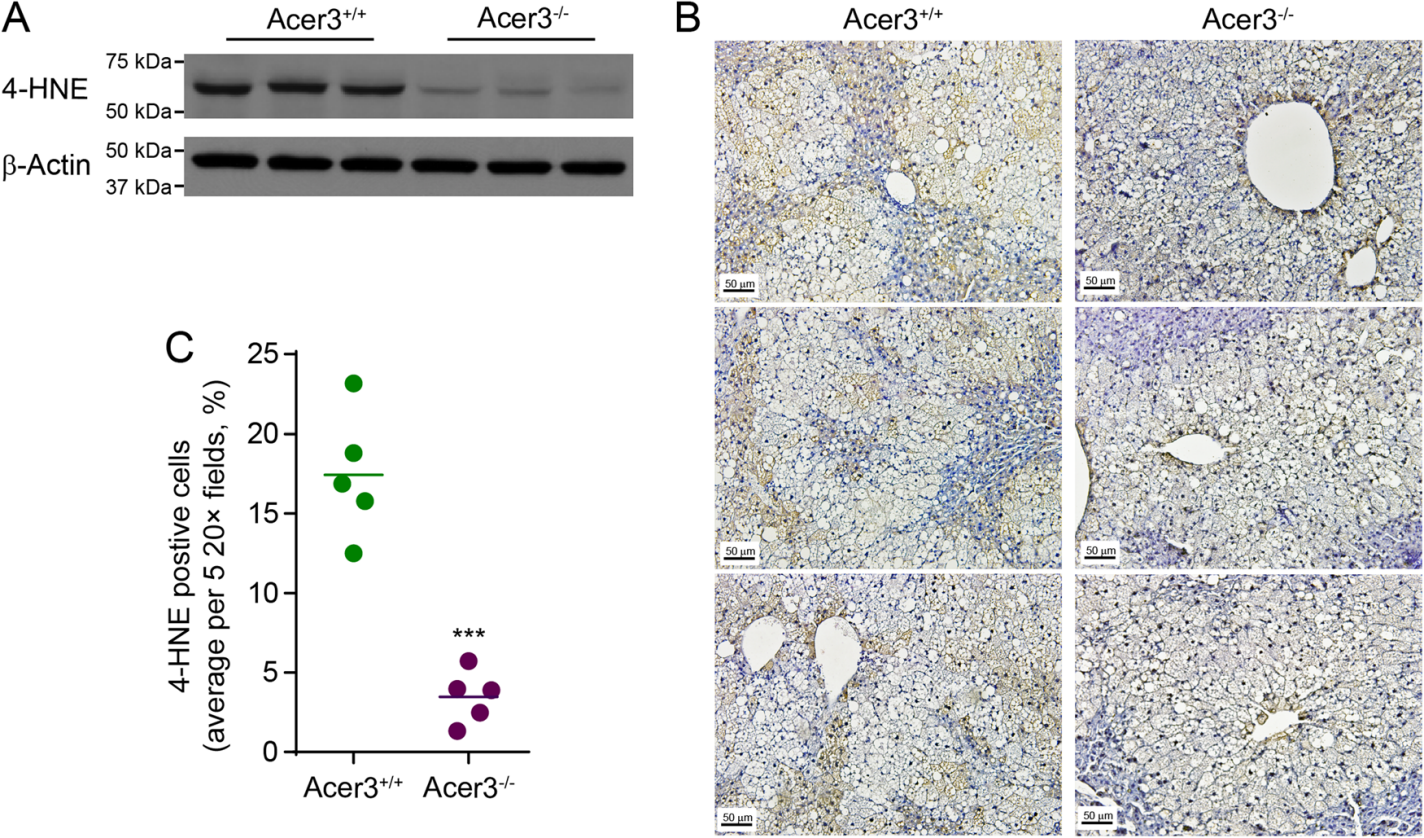
Acer3 deficiency inhibits oxidative stress in hepatocytes in mice with NASH. **a** Liver tissues from Acer3^+/+^ and Acer3^−/−^ mice fed PEWD for 8 weeks were subjected to western blot analyses with the antibody against 4-HNE or β-Actin (a protein loading control). **b** and **c** Liver tissue sections from the above mice were stained with 4-HNE antibody to evaluate oxidative stress **b** and 4-HNE-positive hepatocytes were numerated **c**. Images in **b** represent results from five pairs of mice. ***P < 0.001.

Oxidative stress is a crucial process that induces hepatocellular apoptosis and promotes inflammation during NASH development and progression^34^. As we observed that Acer3 deficiency attenuated both hepatocellular apoptosis and inflammation in NASH liver, we examined if Acer3 deficiency reduces oxidative stress by determining levels of 4-HNE, a lipid peroxidation marker^35^. Indeed, we found that the hepatic levels of 4-HNE were significantly lower in NASH livers of Acer3^−/−^ mice compared to Acer3^+/+^ mice (Fig. 4a). Immunostaining revealed that 4-HNE was detected mostly in steatotic hepatocytes in NASH livers from Acer3^−/−^ and Acer3^+/+^ mice (Fig. 4b) and that the number of hepatocytes positive for staining of 4-HNE was significantly reduced in Acer3^−/−^ mice compared to Acer3^+/+^ mice (Fig. 4c). These data suggest that loss of Acer3 protects hepatocytes from oxidative stress in NASH mice.

### Loss of Acer3 augments palmitic-acid-induced elevation of C_18:1_-ceramide in mouse hepatocytes

**Fig. 5:**
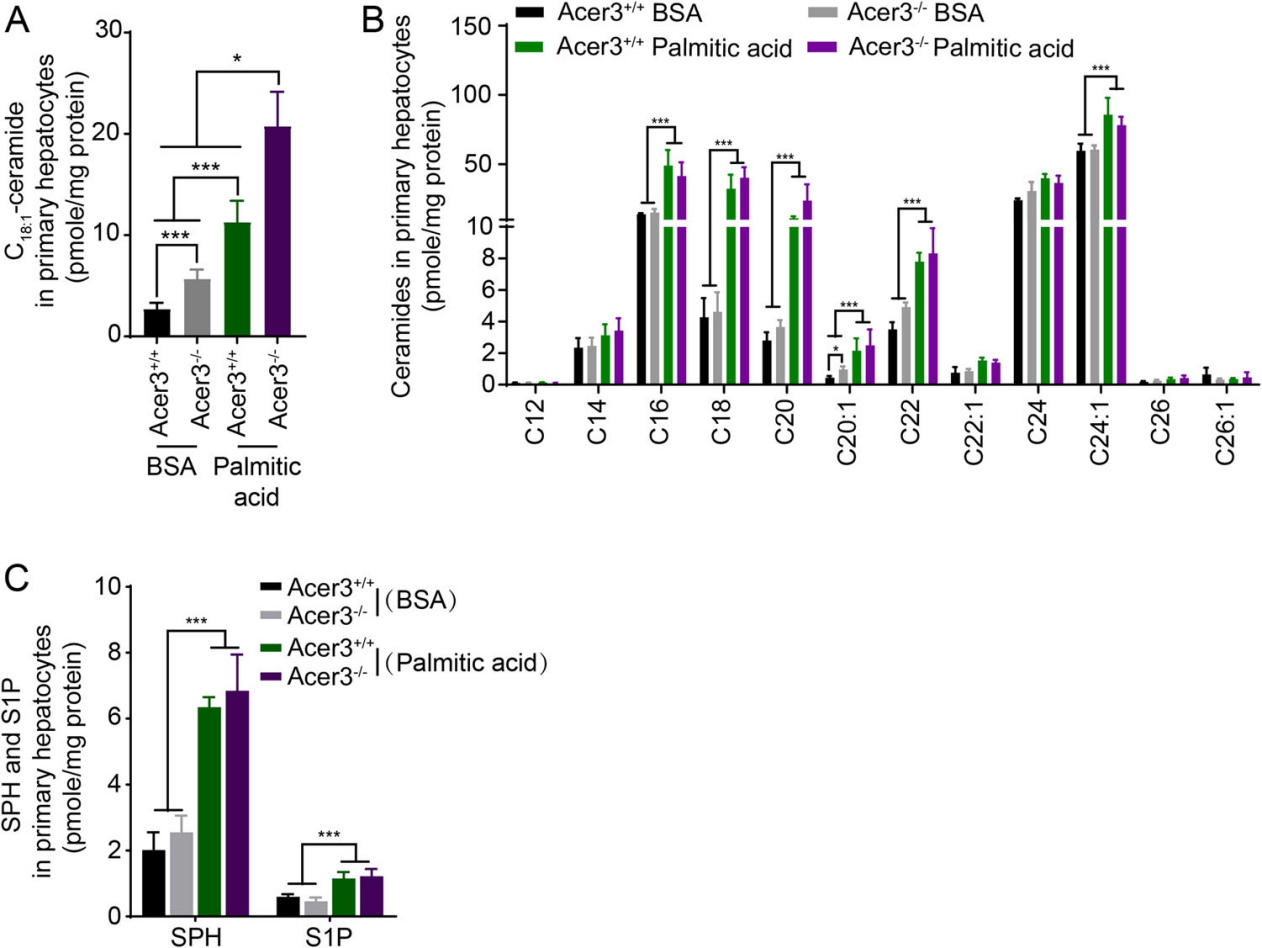
Acer3 deficiency augments an increase in the levels of C_18:1_-ceramide in mouse primary hepatocytes in response to overload of palmitate. Following 6-h treatment with BSA or BSA–palmitate complex (500 μM), LC–MS/MS was performed to analyze the levels of ceramides **a** and **b**, SPH **c**, and S1P **c** in primary hepatocytes from Acer3^+/+^ and Acer3^−/−^ mice. Data represent mean ± SD of three independent experiments. *P < 0.05, ***P < 0.001.

Palmitic acid, which is the most abundant saturated fatty acid in the PEWD used in this study, has been shown to exaggerate NAFLD by stimulating the synthesis of ceramide species mediating cellular damage^7^. To test if loss of Acer3 affected palmitic-acid-induced dysregulation of ceramides in hepatocytes, we isolated primary hepatocytes from Acer3^−/−^ and Acer3^+/+^ mice and treated these cells with palmitate, then determined the levels of ceramides and their metabolites by LC–MS/MS. The results revealed that various long chain ceramides, including C_18:1_-ceramide, were greatly elevated in hepatocytes after palmitate treatment (Fig. 5a, b) and that loss of Acer3 specifically augmented the increase of C_18:1_-ceramide without affecting other ceramide species, SPH or S1P (Fig. 5a, c). These data suggest that palmitic acid indeed increases ceramide levels in hepatocytes, and loss of Acer3 augments palmitic-acid-induced accumulation of the specific ceramide species, C_18:1_-ceramide, in hepatocytes.

### Loss of Acer3 prohibits palmitic-acid-induced apoptosis by suppressing oxidative stress in mouse hepatocytes

**Fig. 6:**
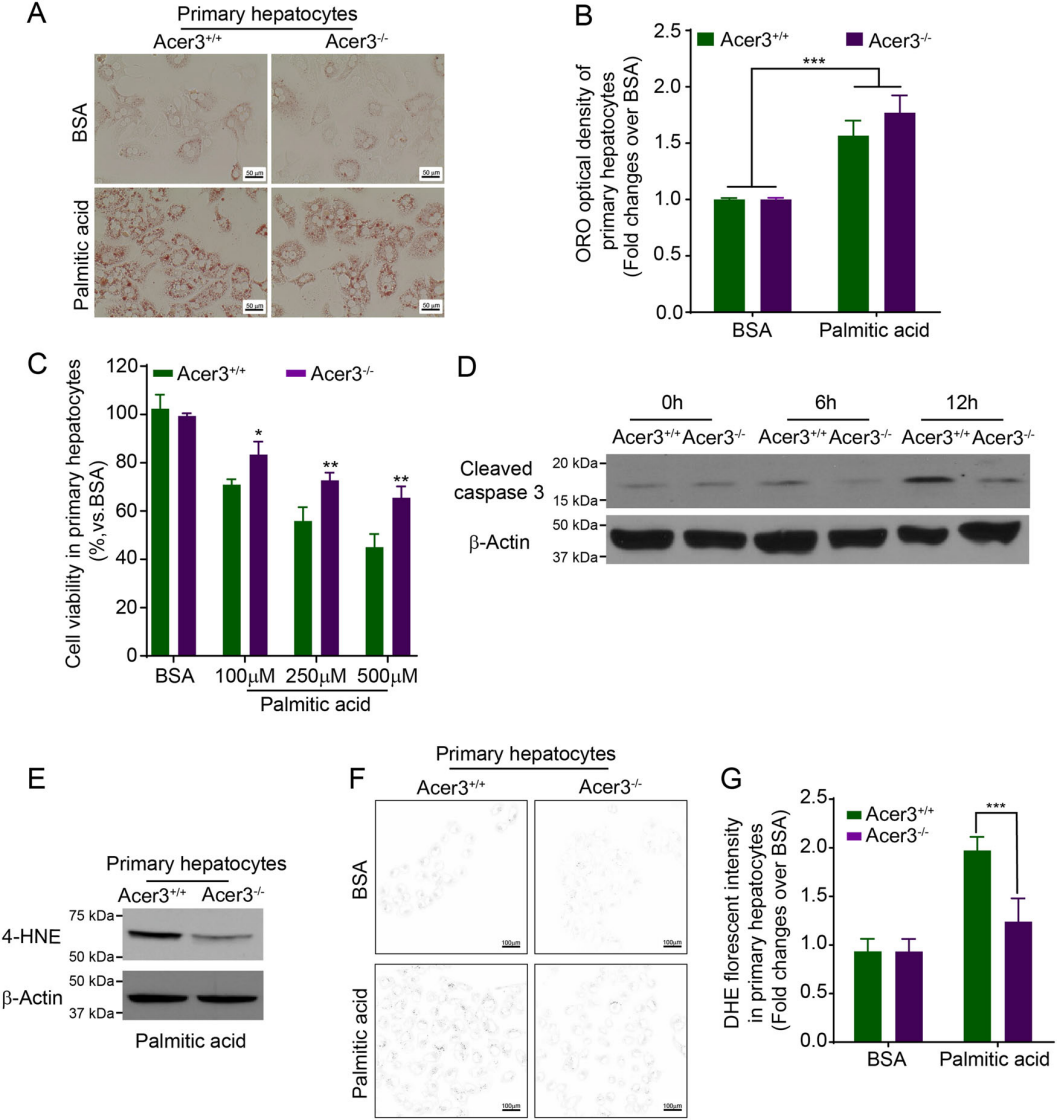
Acer3 deficiency inhibits apoptosis and oxidative stress in mouse hepatocytes in response to overload of palmitate. **a** and **b** Mouse primary hepatocytes treated with BSA or BSA–palmitate complex (500 μM) for 24 h were stained with ORO **a** and the optical density of ORO staining was measured as described in “Materials and methods” section **b**. **c** Hepatocytes were treated with BSA or BSA–palmitate complex at indicated concentrations for 24 h before cell viability was determined by MTT assays as described in “Materials and methods” section. **d** hepatocytes treated with BSA or BSA–palmitate complex (500 μM) for indicated time durations were subjected to Western blot analyses with an antibody against cleaved capase3 or β-Actin (a protein-loading control). **e** Hepatocytes treated with BSA or BSA–palmitate complex (500 μM) for 12 h were subjected to Western blot analyses with the antibody against 4-HNE or β-Actin (a protein-loading control). **f** and **g** DHE staining was performed to measure the production of reactive oxidation species in hepatocytes treated with BSA or BSA–palmitate complex (500 μM) for 12 h. Hepatocytes stained with DHE were imaged by microscopy **g** with background in white and the staining in black, and the florescent intensity was quantified as described in “Materials and methods” section **h**. Data in **b**, **c**, and **g** represent mean ± SD of three independent experiments. Images in **a**, **d**, **e**, and **f** represent results from three independent experiments. *P < 0.05, **P < 0.01, ***P < 0.001.

Ceramides have been reported to regulate the lipotoxicity of palmitic acid that exaggerates NASH by promoting hepatocellular damage^7^. As we found that Acer3 deficiency augmented palmitic-acid-induced elevation of C_18:1_-ceramide, we examined if Acer3 deficiency altered the pro-damage effects of palmitate on hepatocytes. Steatosis induced by palmitate in hepatocytes was examined by ORO staining. We found that there was no difference in steatosis between Acer3^−/−^ and Acer3^+/+^ primary hepatocytes after palmitate treatment (Fig. 6a, b). MTT assays demonstrated that cell viability was reduced to a higher degree in Acer3^+/+^ hepatocytes than in Acer3^−/−^ hepatocytes after palmitate treatment (Fig. 6c). Immunoblotting of apoptotic marker proteins further demonstrated that loss of Acer3 inhibited an increase in the levels of cleaved caspase 3 in hepatocytes after treatment of palmitate (Fig. 6d). These data suggest that loss of Acer3 alleviates palmitic-acid-induced apoptosis in hepatocytes.

Oxidative stress is an important mechanism by which palmitic acid triggers hepatocellular apoptosis, leading to inflammation and fibrosis in NASH progression^34^. Having demonstrated that Acer3 knockout alleviated both apoptosis and oxidative stress in hepatocytes in NASH livers and protected hepatocytes in culture from palmitate-induced apoptosis, we tested whether loss of Acer3 inhibited palmitate-induced oxidative stress in hepatocytes. Immunoblotting of 4-HNE revealed that Acer3 deficiency downregulated levels of 4-HNE in palmitic-acid-treated murine hepatocytes (Fig. 6e). Production of reactive oxygen species after palmitate treatment was measured by DHE staining. We found that Acer3 deficiency reduced staining intensity of DHE in hepatocytes after palmitate treatment (Fig. 6f, g), suggesting that loss of Acer3 indeed prohibits the production of reactive oxygen species in hepatocytes upon palmitate challenge. In conclusion, these data demonstrated that loss of Acer3 attenuates oxidative stress triggered by palmitate in hepatocytes.

### Knocking down ACER3 elevates C_18:1_-ceramide and protects human hepatocytes against palmitic-acid-induced apoptosis and oxidative stress

**Fig. 7:**
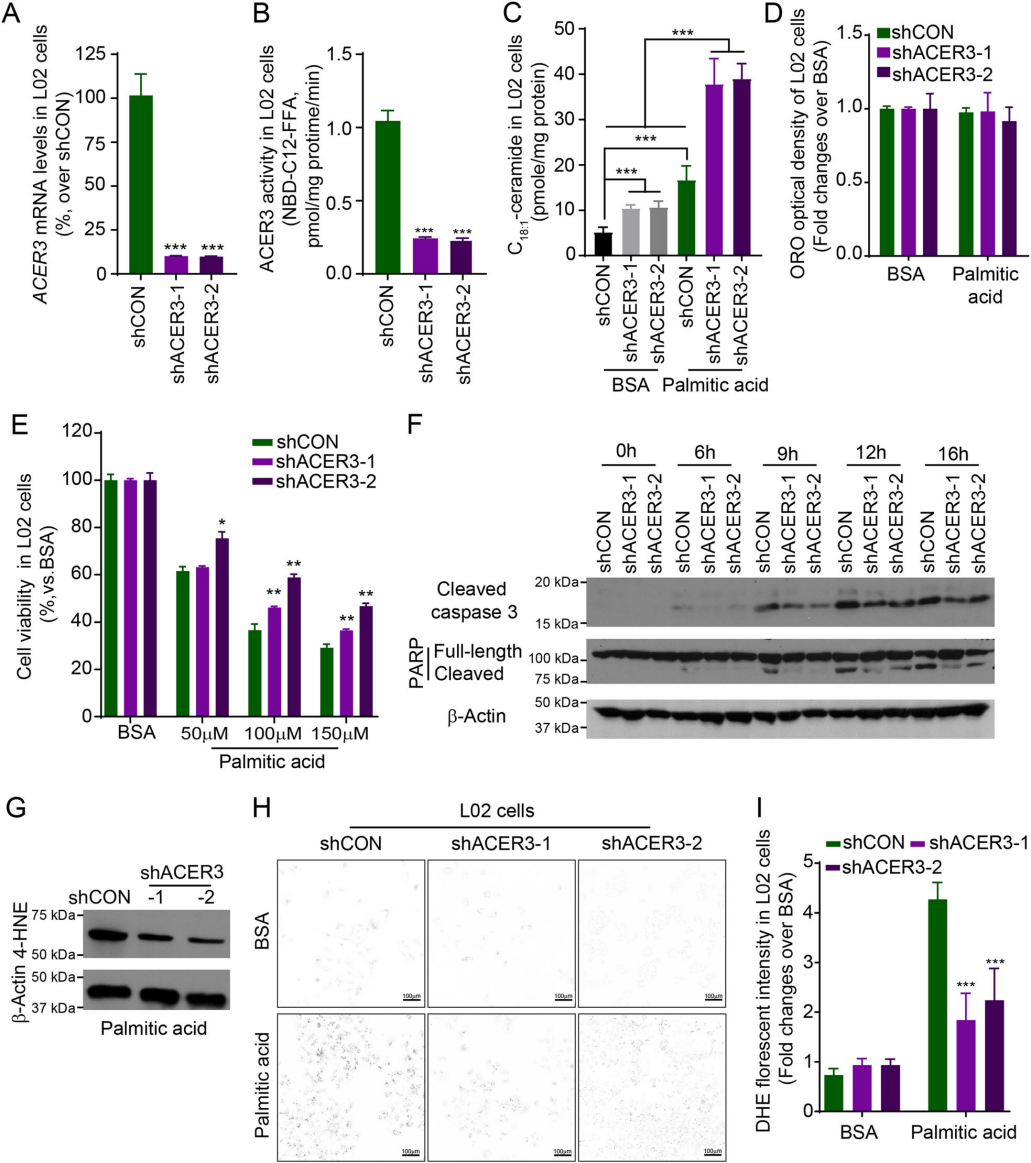
Knocking down ACER3 elevates C_18:1_-ceramide and protects human hepatocytes from apoptosis and oxidative stress in response to overload of palmitate. **a** and **b** Human L02 hepatocytes were transfected with each of two short hairpin RNAs (shRNA) specific for the *ACER3* gene (shACER3-1 and shACER3-2) or a control shRNA (shCON), ACER3 mRNA **a** and enzymatic activity **b** were measured by qPCR and in vitro activity assays, respectively. **c** L02 cells transfected with shACER3-1, shACER3-2, or shCON were treated with BSA or BSA–palmitate complex (100 μM) for 6 h before the levels of ceramides were determined by LC–MS/MS. **d** and **e** L02 cells transfected with shACER3-1, shACER3-2, or shCON were treated with BSA or BSA–palmitate complex (100 μM) for 24 h before ORO staining **d** or MTT assays were performed **e**. The optical density of ORO staining was measured as described in “Materials and methods” section. **f** L02 cells transfected with shACER3-1, shACER3-2, or shCON were treated with BSA or BSA–palmitate complex (100 μM) for indicated time durations before western blot analyses were performed with an antibody against PARP, cleaved caspase 3, or β-Actin (a protein-loading control). **g** L02 cells transfected with shACER3-1, shACER3-2, or shCON were treated with BSA or BSA–palmitate complex (100 μM) for 12 h before Western blot analyses were performed with the antibody against 4-HNE or β-actin (a protein-loading control). **h** and **i** L02 cells transfected with shACER3-1, shACER3-2, or shCON were treated with BSA or BSA–palmitate complex (100 μM) for 12 h before DHE staining was imaged **h** with background in white and the staining in black, and the florescent intensity was detected as described in “Materials and methods” section **i**. Data in **a**–**e** and **i** represent results mean ± SD of three independent experiments. Images in **f**–**h** represent results from three independent experiments. *P < 0.05, **P < 0.01, ***P < 0.001.

To consolidate the notion that ACER3 upregulation mediates palmitate-induced hepatocellular injury and oxidative stress, we investigated whether the human ACER3 has similar pathological roles in palmitate-induced apoptosis and oxidative stress of hepatocytes. To this end, we first established two human hepatocyte L02 lines (L02-shACER3-1 or L02-shACER3-2) in which *ACER3* were stably knocked down by ACER3-specific short hairpin RNAs (shACER3-1 and shACER3-2). qPCR and ACER3 activity assays confirmed that ACER3 expression was markedly suppressed in L02-shACER3-1 or L02-shACER3-2 cells compared to the control L02 cell line (L02-shCON) expressing a control shRNA (shCON) (Fig. 7a, b). LC–MS/MS showed that knockdown of ACER3 markedly enhanced palmitate-induced increase in the levels of C_18:1_-ceramide, but not other ceramide species in L02 cells (Fig. 7c, Supplementary Figs. S2A and S2B). Steatosis in L02 cell was not affected by knockdown of ACER3 after palmitate treatment (Fig. 7d). MTT assays demonstrated that ACER3 knockdown inhibited palmitate-induced loss of viability of L02 cells (Fig. 7e). Immunoblotting analysis found that ACER3 knockdown inhibited palmitate-induced PARP and caspase 3 cleavage in L02 cells (Fig. 7f). ACER3 knockdown also decreased the levels of 4-HNE (Fig. 7g) and reduced the staining of DHE (Fig. 7h, i) in L02 cells after palmitate treatment. These results consolidate that human ACER3 upregulation mediates palmitic-acid-induced hepatocellular injury via oxidative stress in the context of NASH.

## Discussion

In this study, we demonstrate for the first time that the ceramide-degrading enzyme ACER3 is upregulated in NASH livers and that its upregulation contributes to the pathogenesis of Western-diet-induced NASH by exacerbating hepatocellular injury in response to oversupply of saturated fatty acids. We provided evidence that ACER3 upregulation mediates the pathogenesis of NASH by reducing the hepatic levels of C_18:1_-ceramide, which protects hepatocytes from oxidative stress in response to overload of palmitic acid.

Aiming at elucidating the dysregulation of ceramidases responsible for the degradation of ceramides in NASH liver, we performed in silico analyses on mRNA levels of genes involved in sphingolipid metabolism using a NCBI GEO dataset published by Markus Ahrens et al.^32^. The mRNA levels of *ACER3* were significantly upregulated in the NASH liver compared to the NAFL or healthy liver (Fig. 1). Consistent with these human data, we found that the mouse Acer3 was also significantly upregulated in the liver with NASH that was induced by PEWD (Fig. 1). Moreover, we demonstrated that oversupply of palmitate but not oleic acid substantially upregulated the mouse Acer3 in hepatocytes (Fig. 1). Since palmitate is the most abundant saturated fatty acid in serum of NAFL patients and its serum levels are further increased in NASH patients^5,36^, we postulated that the overload of palmitate may result in the upregulation of Acer3/ACER3 in hepatocytes during progression of NAFL to NASH.

In this study, we demonstrated that Acer3 knockout protects hepatocytes from apoptosis in NASH livers, suggesting that Acer3 mediates hepatocyte death in NASH (Figs. 3, 6, and 7). By analyzing the effects of Acer3 knockout or ACER3 knockdown on the metabolism of ceramides in hepatocytes, we found that ACER3 upregulation prevents the buildup of the C_18:1_-ceramide in diet-induced NASH livers and palmitic-acid-treated hepatocytes. This is consistent with the fact that either mouse Acer3 or human ACER3 preferentially catalyzes the hydrolysis of unsaturated ceramide species, C_18:1_-ceramide in particular^19,29,30^. These results suggest that C_18:1_-ceramide protects hepatocytes from palmitic acid-induced apoptosis. Interestingly, we previously found that Acer3 has a protective role in survival of neurons. Acer3 deficiency resulted in an increase in the levels of C_18:0_-ceramide in mouse brain, in addition to C_18:1_-ceramide, suggesting that C_18:1_-ceramide and C_18:0_-ceramide may have opposing roles in regulating cell survival and death, with the former being a pro-survival lipid and the latter a pro-death lipid. The difference in the relative levels of specific ceramide species between hepatocytes and neurons may explain why ACER3 promotes survival of one cell type while mediating death of another cell type. Indeed, although previous studies have suggested that all ceramide species, as a whole, are pro-apoptotic bioactive lipids^37,38^, emerging studies have demonstrated that certain ceramide species promote cell survival rather than inducing cell death^39,40^.

Oversupply of palmitate has been shown to trigger oxidative stress in NASH liver^41,42^. Oxidative stress was ameliorated in hepatocytes from Acer3 null mice fed PEWD (Fig. 4). Consistently, we further revealed that loss of Acer3 or knockdown of ACER3 prohibited palmitic-acid-induced oxidative stress in hepatocytes in culture (Figs. 5 and 6), suggesting that C_18:1_-ceramide buildup inhibits the oxidative stress in hepatocytes in response to overload of palmitic acid. Interestingly, Hila Zigdon et al. reported that C_16_-ceramide promotes the generation of reactive oxygen species by inhibiting complex IV activity in mitochondria, resulting in chronic oxidative stress^43^, demonstrating that C_18:1_-ceramide and C_16_-ceramide may have opposing roles.

Oxidative stress is a critical mechanism of hepatocyte apoptosis and inflammatory in NASH liver^44^. The production of reactive oxygen species upon oxidative stress is reported to cause both nuclear and mitochondrial DNA damage and eventually induce hepatocyte death^45^. Moreover, reactive oxygen species along with products of lipid peroxidation also increase levels of pro-inflammatory cytokines including TNF-α, interleukin 1, and IL-6, which play an important role in leukocytes infiltration and macrophage activation^46^. These results suggest that the inhibition of oxidative stress by Acer3 deficiency contributes to the alleviation of early hepatocyte apoptosis, inflammation, and fibrosis in NASH liver.

In conclusion, we for the first time demonstrate that ACER3 has a pathological role in the progression of PEWD-induced NASH by regulating the hepatic levels of C_18:1_-ceramide that may counteract the effects of palmitate on oxidative stress in hepatocyte. This study also suggests that targeting ACER3 represents a novel approach to prevention and intervention of NASH.

## Supplementary information

SUPPLEMENTARY INFORMATION

Figure S1

Figure S2

Table S1
