# Correction: Targeting alkaline ceramidase 3 alleviates the severity of nonalcoholic steatohepatitis by reducing oxidative stress

**DOI:** 10.1038/s41419-020-2396-1

**Published:** 2020-03-17

**Authors:** Kai Wang, Chuanjiang Li, Xinxin Lin, Hang Sun, Ruijuan Xu, Qingping Li, Yiran Wei, Yiyi Li, Jianping Qian, Cuiting Liu, Qifan Zhang, Sheng Yu, Zhonglin Cui, Xixin Huang, Bili Zhu, Jie Zhou, Cungui Mao

**Affiliations:** 1 Division of Hepatobiliopancreatic Surgery, Department of General Surgery, Nanfang Hospital, Southern Medical University, Guangzhou, Guangdong China; 2 Department of Medicine and Cancer Center, the State University of New York at Stony Brook, Stony Brook, New York, USA; 3 The First Clinical College, Southern Medical University, Guangzhou, Guangdong China; 4 Department of Radiation Oncology, Nanfang Hospital, Southern Medical University, Guangzhou, Guangdong China; 5 Central Laboratory, Southern Medical University, Guangzhou, Guangdong China; 6 Huiqiao Building, Nanfang Hospital, Southern Medical University, Guangzhou, Guangdong China

**Keywords:** Lipidomics, Non-alcoholic steatohepatitis

**Correction to: Cell Death & Disease**

10.1038/s41419-019-2214-9, published online 16 January 2020

In the original published version of the article, there was a mistake in the affiliations:

The first affiliation (“Department of Hepatobiliary Surgery, Nanfang Hospital, Southern Medical University, Guangzhou, Guangdong, China”) should read “Division of Hepatobiliopancreatic Surgery, Department of General Surgery, Nanfang Hospital, Southern Medical University, Guangzhou, Guangdong, China”

This has been corrected in both the PDF and HTML versions of the Article.

