# Supplementary material for: Targeting alkaline ceramidase 3 alleviates the severity of nonalcoholic steatohepatitis by reducing oxidative stress
Source: Cell Death Dis. 2020 Jan 16;11(1):28. doi: 10.1038/s41419-019-2214-9 (PMC6965144; doi:10.1038/s41419-019-2214-9)
Supplement: Supplementary file 1 — SUPPLEMENTARY INFORMATION [file 41419_2019_2214_MOESM1_ESM.docx]

**SUPPLEMENTARY INFORMATION**

**Figure S1. Dihydroceramide desaturase, sphingomyelinases and sphingomyelin synthase are dysregulated in the livers of patients with NAFLD**

The NCBI GEO database, GSE48452, was analyzed for the mRNA levels of genes encoding sphingolipid-metabolizing enzymes in liver tissues from 14 healthy individuals, 14 NAFL patients, and 18 NASH patients. The mRNA levels of the sphingomyelinases, *SMPD2* and *SMPD3*, were increased in NASH livers compared to healthy livers while the opposite is true with the desaturase *DEGS1* and sphingomyelin synthase *SMS2*. **P*<0.05.

**Figure S2. ACER3 knockdown does not affect increases in the levels of saturated long-chain ceramides, very long-chain ceramides, SPH, and S1P in human L02 hepatocytes in response to overload of palmitate**

Following 6-hour treatment with BSA or BSA-palmitate complex (100 μM), LC-MS/MS was performed to analyze the levels of ceramides (**A**), SPH (**B**), and S1P (**B**) in L02 hepatocytes transfected with shCON, shACER3-1, or shACER3. Data represent mean ± SD of 3 independent experiments. **P*<0.05, P*<0.01, and ****P*<0.001.

**Table S1**. **Information of qPCR primer sequence used in this study.**
