# Supplementary figures and images for: Targeting alkaline ceramidase 3 alleviates the severity of nonalcoholic steatohepatitis by reducing oxidative stress
Source: Cell Death Dis. 2020 Jan 16;11(1):28. doi: 10.1038/s41419-019-2214-9 (PMC6965144; doi:10.1038/s41419-019-2214-9)

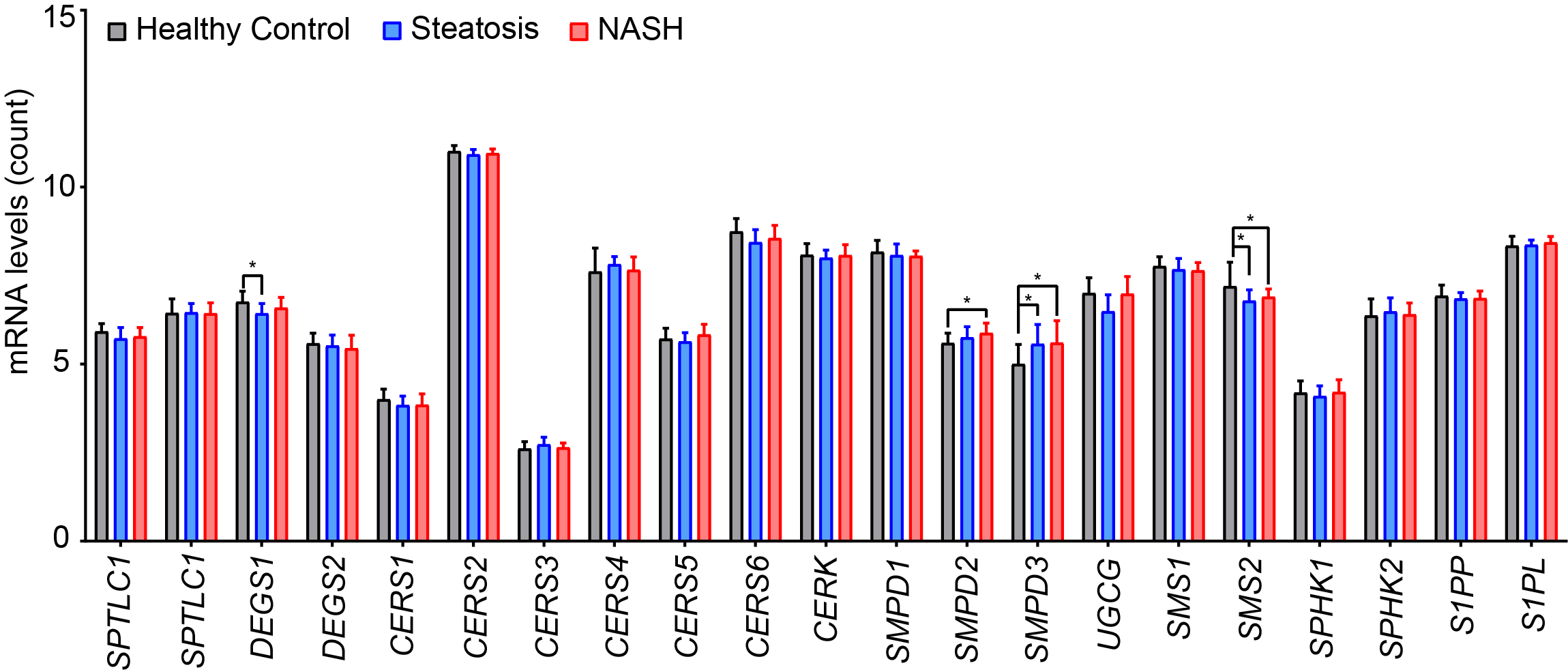

Supplement: Supplementary file 2 — Figure S1 [file 41419_2019_2214_MOESM2_ESM.png]

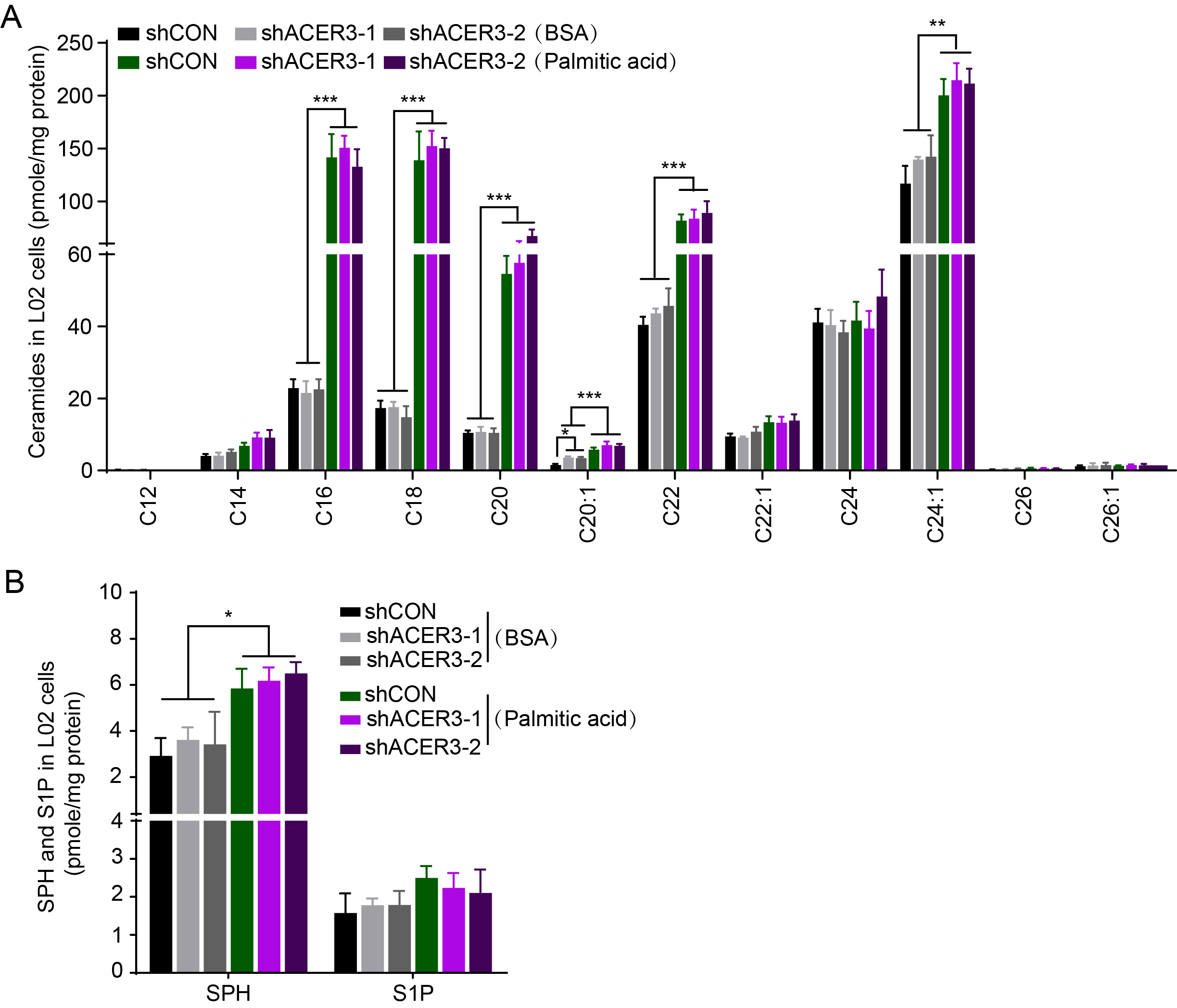

Supplement: Supplementary file 3 — Figure S2 [file 41419_2019_2214_MOESM3_ESM.png]
