# Supplementary material for: Targeting alkaline ceramidase 3 alleviates the severity of nonalcoholic steatohepatitis by reducing oxidative stress
Source: Cell Death Dis. 2020 Jan 16;11(1):28. doi: 10.1038/s41419-019-2214-9 (PMC6965144; doi:10.1038/s41419-019-2214-9)
Supplement: Supplementary file 4 — Table S1 [file 41419_2019_2214_MOESM4_ESM.docx]

| **Gene** | **qPCR primer sequence** |
| --- | --- |
| *Acer3* | 5’- GATTCACTGAGGAACTTTCG -3’  5’- AGAGAAACTTCACTTTTGGC -3’ |
| *ACER3* | 5’ -GACTGGGTTATACATCATTGG-3’  5’-CCTCAGTGACTCACAAAATATG-3’ |
| *β−Actin* | 5’-TGTGCACTTTTATTGGTCTC-3’  5’-GATGTATGAAGGCTTTGGTC-3’ |
| *β−ACTIN* | 5’ -CAATGTTCGGTGCAATTCAGAG-3’  5’- CGGTTCCGATGCCCTGAGGCTCTT-3’ |
| *Il-6* | 5’ -AAGAAATGATGGATGCTACC-3’  5’- GAGTTTCTGTATCTCTCTGAAG-3’ |
| *Tnf-α* | 5’- CATCTTCTCAAAATTCGAGTGACAA-3’  5’- TGGGAGTAGACAAGGTACAACCC-3’ |
| *Tgf-β* | 5’-CCCTATATTTGGAGCCTGGA-3’  5’ -CTTGCGACCCACGTAGTAGA-3’ |

Table S1. Information of qPCR primer sequence.
